# Prevalence and molecular insights into carbapenem resistance: a 2-year retrospective analysis of superbugs in South India

**DOI:** 10.3389/fmed.2025.1571231

**Published:** 2025-05-30

**Authors:** Rahul Harikumar Lathakumari, Leela Kakithakara Vajravelu, Jayaprakash Thulukanam, Dakshina M. Nair, Poornima Baskar Vimala, Vishnupriya Panneerselvam

**Affiliations:** Department of Microbiology, SRM Medical College Hospital and Research Centre, SRM Institute of Science and Technology (SRMIST), Kattankulathur, Chengalpattu, Tamil Nadu, India

**Keywords:** carbapenem resistance, molecular characterization, hospital acquired infections, imipenem, meropenem

## Abstract

**Background:**

Carbapenem-resistant Gram-negative bacteria (CR-GNB) pose a serious global health threat, especially in low- and middle-income countries. Local surveillance is crucial for informing antimicrobial stewardship and infection control strategies. This study aimed to evaluate the prevalence, demographic distribution, and temporal fluctuations of carbapenem resistance among key Gram-negative pathogens in a South Indian tertiary care center over a two-year period.

**Methods:**

A retrospective study was conducted on 8,359 non-duplicate Gram-negative isolates obtained from clinical specimens between July 2022 and July 2024. Organisms were identified, and antimicrobial susceptibility was determined using the VITEK^®^ 2 Compact system (BioMérieux). Resistance to imipenem (IPM) and meropenem (MEM) was assessed. Data were stratified by age, sex, ward type, specimen source, and quarterly distribution. A subset of resistant isolates underwent molecular screening for carbapenemase genes using real time PCR.

**Results:**

Carbapenem resistance was observed in 24% (2007) of Gram-negative isolates. *Acinetobacter baumannii* (48.0%) and *Klebsiella pneumoniae* (38.6%) accounted for the majority of resistant cases. Resistance was significantly higher in males (64.3%) and in patients aged 61–80 years (*p* < 0.001). Surgical wards showed greater resistance rates compared to medical departments. A peak in resistance was identified during January–March 2023, particularly for *A. baumannii* (76.3%). IPM-MEM resistance discrepancies were found in *Citrobacter* and *Proteus* species. Gene profiling of resistant strains revealed the predominance of *bla*_NDM_, *bla*_VIM_ in *all organism.*

**Conclusion:**

The findings reveal a high and fluctuating burden of carbapenem resistance, especially in elderly males and surgical settings. Continuous surveillance and targeted interventions are vital to curbing the spread of CR-GNB in high-risk healthcare environments.

## 1 Introduction

Carbapenem-resistant organisms (CROs) represent a significant and growing challenge in healthcare settings worldwide (1). These organisms are often associated with high morbidity, mortality, and healthcare costs due to limited treatment options and the potential for widespread outbreaks. Carbapenems, a class of *β*-lactam antibiotics with a broad spectrum of activity, have historically been considered drugs of last resort for treating severe infections caused by multi-drug-resistant GNB (2). However, the emergence and spread of carbapenem resistance have severely compromised their effectiveness (3).

The mechanisms of carbapenem resistance are diverse, including the production of carbapenemase enzymes (e.g., KPC, NDM, VIM, IMP, and OXA types), efflux pumps, and porin mutations that reduce antibiotic uptake (4). The spread of these resistant organisms is facilitated by plasmids and other mobile genetic elements that can be transferred between bacteria, accelerating the dissemination of resistance genes across different species and geographical regions (5, 6).

In India, the prevalence of carbapenem-resistant organisms is particularly concerning. Studies have reported high rates of carbapenem resistance among key pathogens such as *K. pneumoniae, A. baumannii, and Pseudomonas aeruginosa* (7). The National Antimicrobial Resistance Surveillance Network (NARS-Net) in India has highlighted alarming levels of resistance, with reports indicating that over 50% of *K. pneumoniae* isolates and 30% of *P. aeruginosa* isolates are resistant to Carbapenems. These figures underscore the critical need for enhanced surveillance, stringent infection control measures, and the development of new therapeutic strategies.

Tertiary care centre, which provide specialized medical care and serve as referral hospitals, are particularly vulnerable to the spread of CROs (8). Patients in these settings often have complex medical conditions, require invasive procedures, and are exposed to broad-spectrum antibiotics, all of which increase the risk of acquiring resistant infections. Monitoring the prevalence of carbapenem resistance in such settings is crucial for developing effective infection control strategies and guiding empirical therapy (9).

In this study, we conducted a retrospective analysis of carbapenem resistance in a tertiary care centre over a two-year period. Our objectives were to determine the prevalence of CROs, identify trends over time, and evaluate the distribution of resistance among different bacterial species. In addition to phenotypic analysis, we also performed molecular characterization to identify the specific resistance genes responsible for carbapenem resistance. By analysing these data, we aim to provide insights that can inform clinical practice and policy decisions to better manage and prevent the spread of carbapenem-resistant infections.

## 2 Materials and methods

### 2.1 Study design, setting, and population

This retrospective study was conducted to evaluate the prevalence and trends of CROs over a two-year period, from July 2022 to July 2024, at SRM Medical College Hospital and Research Centre, SRMIST, Kattankulathur, Chennai, Tamil Nadu. The study included all patients from whom clinical specimens were submitted for microbiological analysis during the study duration. Various specimen types—including blood, urine, respiratory secretions, and exudates—were processed for bacterial culture and antimicrobial susceptibility testing. All patients, irrespective of age or gender, were considered eligible, provided their samples yielded growth of GNB. Specimens that showed no bacterial growth, yielded Gram-positive organisms, or represented duplicate isolates from the same patient were excluded to avoid duplication and ensure the accuracy of the prevalence data.

### 2.2 Antimicrobial susceptibility testing and data collection

AST was performed using the VITEK system (BioMérieux), an advanced automated method for bacterial identification and susceptibility pattern. The panel of antibiotics tested against the CROs included AN, Amikacin; ATM, Aztreonam; CAZ, Ceftazidime; CIP, Ciprofloxacin; CS, Colistin; FEP, Cefepime; FOS, Fosfomycin; GM, Gentamicin; LEV, Levofloxacin; MNO, Minocycline; SXT, Trimethoprim/Sulfamethoxazole; TZP, Piperacillin/Tazobactam along with carbapenems such as Imipenem (IPM) and Meropenem (MEM). These antibiotics were selected based on CLSI guidelines to cover a broad spectrum of resistance mechanisms found in Enterobacterales, *Pseudomonas,* and *Acinetobacter* (10). The resistance profiles for IPM and MEM were determined separately using the same VITEK AST-N405 and VITEK AST-N406 cards that were used to test all antibiotics for GNB.

An isolate was classified as carbapenem-resistant if it exhibited resistance to either or both drugs, regardless of its susceptibility to ertapenem, in accordance with CLSI recommendations. For Enterobacteriaceae, resistance was defined as a MIC value of ≥4 μg/mL for IPM or MEM. For *P. aeruginosa* and *A. baumannii*, resistance was defined as a MIC value of ≥8 μg/mL for these antibiotics.

### 2.3 Molecular characterization

Genomic DNA was extracted from pure cultures of carbapenem-resistant isolates grown overnight on nutrient agar plates. Bacterial cells were transferred into centrifuge tubes containing sterile double-distilled water, boiled at 95°C for 15 min, centrifuged at 15000 rpm for 10 min and stored at −72°C. Plasmid DNA was isolated using Truescreen magnetic bead-based extraction kit (developed by TranScience innovative Technologies Pvt. Ltd.) after lysis with Proteinase K. DNA binding to magnetic beads was facilitated by Truescreen solution, and the bound plasmid DNA was separated from cellular debris through magnetic separation and washing. Primers targeting carbapenemase genes *bla*_VIM_*, bla*_NDM_*, and bla*_OXA-48_ were designed based on ICMR guidelines and synthesized by Eurofins Genomics (Table 1). A SYBR Green-based master mix containing AmpliTaq Gold DNA polymerase and optimized components was prepared with primers and template DNA. Real-time PCR (ABI PRISM 7900HT) was conducted for absolute quantification, with an amplification protocol consisting of initial denaturation at 95°C, followed by cycles of denaturation, annealing, and extension. Genotypically confirmed positive ATCC control strains, obtained from HiMedia (India), were included in each PCR run to validate the assay. These strains were not sequenced, as their resistance genotypes are well characterized. This confirmed the presence of the *bla*_NDM_*, bla*_OXA-48_*, and bla*_VIM_ genes, identifying the molecular basis of carbapenem resistance in these isolates (5).

**Table 1:** Primer sequences and amplicon sizes for detection of carbapenem resistance genes by PCR.

Resistance gene	Forward primer	Reverse primer	Amplicon length
*bla*_VIM_	GATGGTGTTTGGTCGCATA	CGAATGCGCAGCACCAG	390 bp
*bla*_NDM_	CCGTATGAGTGATTGCGGCG	GCCCAATATTATHCACCCGG	779 bp
*bla*_OXA-48_	GCTTGATCGCCCTCGATT	GATTTGCTCCGTGGCCGAAA	570 bp

### 2.4 Statistical analysis

Data were analysed using IBM SPSS Statistics version 27 to determine the prevalence and trends of carbapenem resistance. Descriptive statistics were employed to summarize patient demographics, specimen types, bacterial species, and resistance profiles. The prevalence of CROs was calculated as the proportion of resistant isolates relative to the total number of GNB isolates. Categorical data were described using frequency and percentages and analysed using Chi square test. A *p* < 0.05 was considered statistically significant.

### 2.5 Ethical considerations

Ethical approval was obtained from the Institutional Ethics Committee of SRMMCH&RC. Given the retrospective nature of the study, individual patient consent was not required. However, patient confidentiality was strictly maintained, and data were anonymized prior to analysis to protect patient privacy.

## 3 Results

### 3.1 Prevalence of carbapenem-resistance in GNB

**Figure 1:**
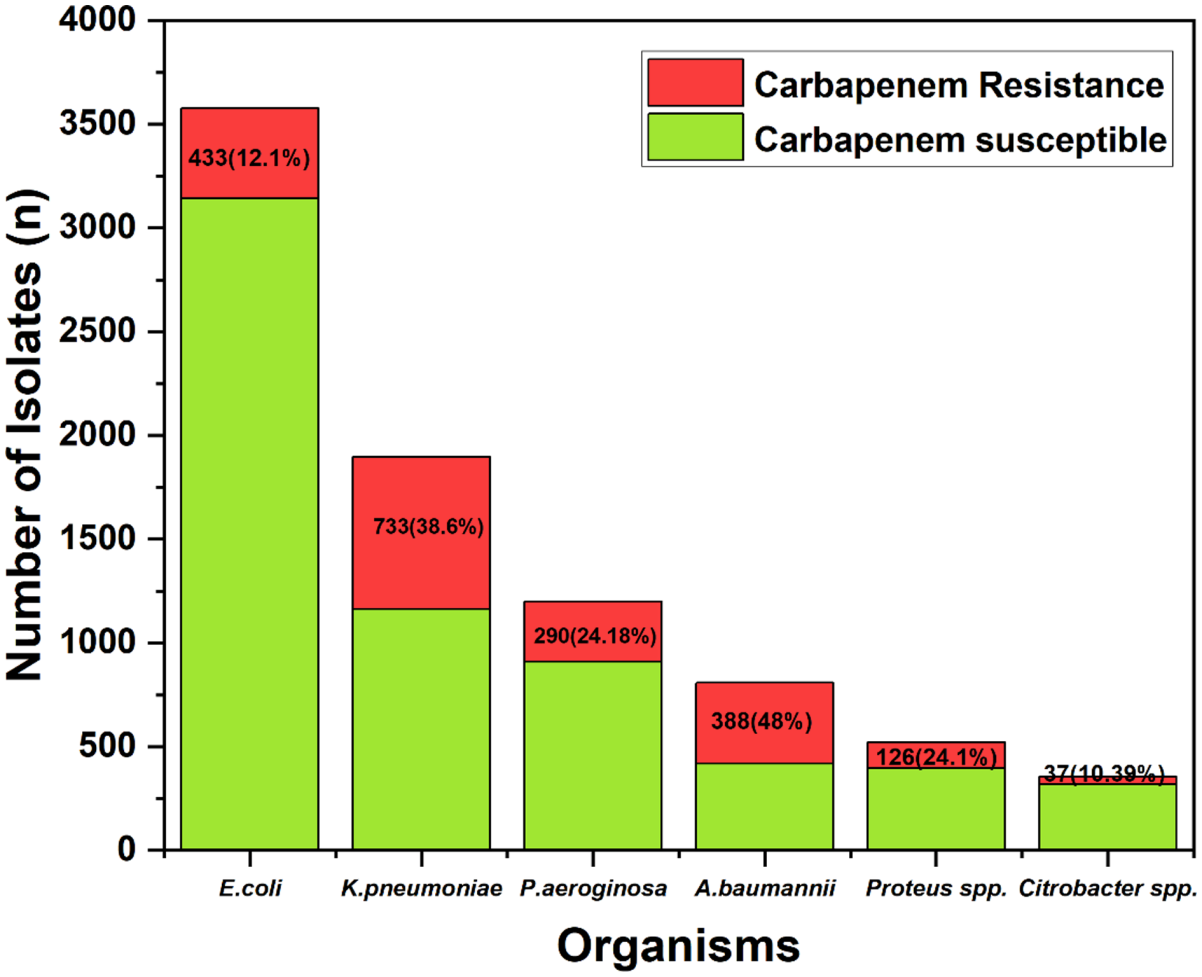
Prevalence of carbapenem resistance in GNB, showing highest resistance in *A. baumannii* (48%) and lowest in *Citrobacter* spp. (10.39%).

During the study period from July 2022 to July 2024, a total of 8,359 clinical specimens yielded growth of GNB and were included in the analysis. The overall prevalence of carbapenem-resistant GNB among the isolates was significant. *A. baumannii* exhibited the highest level of resistance at 48% (388 out of 808 isolates), followed by *K. pneumoniae* at 38.6% (733 out of 1,897 isolates). *P. aeruginosa* showed a resistance rate of 24.18% (290 out of 1,199 isolates), while *Proteus* spp. had a resistance rate of 24.1% (126 out of 522 isolates). The prevalence of carbapenem resistance in *E. coli* was 12.1% (433 out of 3,577 isolates), and *Citrobacter* spp. showed the lowest resistance rate at 10.39% (37 out of 356 isolates). These findings (Figure 1) highlight the significant burden of carbapenem resistance among GNB in the tertiary care centre.

To further evaluate the association between organism type and carbapenem resistance, a Chi-square test was performed. The test revealed a statistically significant association (*p* = 0.019*), with *A. baumannii* and *K. pneumoniae* emerging as the organisms most strongly associated with carbapenem resistance.

### 3.2 Carbapenem resistance across clinical specimens and hospital alliances

**Figure 2:**
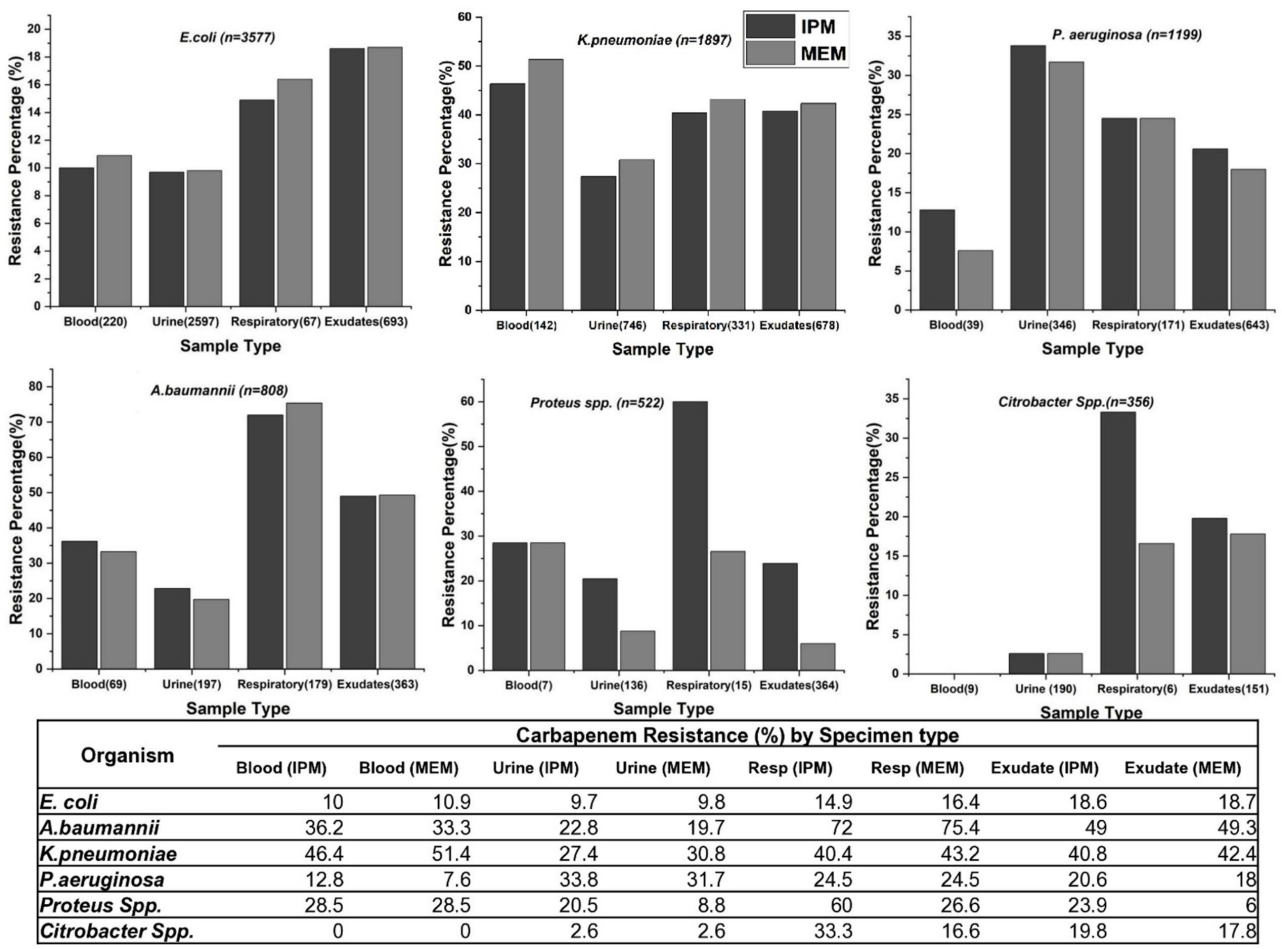
Carbapenem resistance rates in various clinical specimens for different bacterial species. The graphs display IPM and MEM resistance rates across blood, urine, respiratory samples, and exudates for *E. coli*, *K. pneumoniae*, *P. aeruginosa*, *A. baumannii*, *Citrobacter* spp. *and Proteus* spp.

The study examined carbapenem resistance patterns in key GNB—*E. coli, K. pneumoniae, P. aeruginosa, A. baumannii, Proteus* spp., and *Citrobacter* spp.—across various clinical specimens and hospital alliances. Overall, *K. pneumoniae* and *A. baumannii* exhibited the highest resistance to both IPM and MEM, particularly in blood, respiratory and exudate samples. Among *K. pneumoniae* isolates, those from blood samples exhibited the highest carbapenem resistance (51.4%), whereas no *Citrobacter* spp. were isolated from blood. In *E. coli*, isolates from exudates showed the highest resistance (>18%), while *Proteus* spp. and *Citrobacter* spp. displayed greater resistance in respiratory samples (Figure 2).

**Figure 3:**
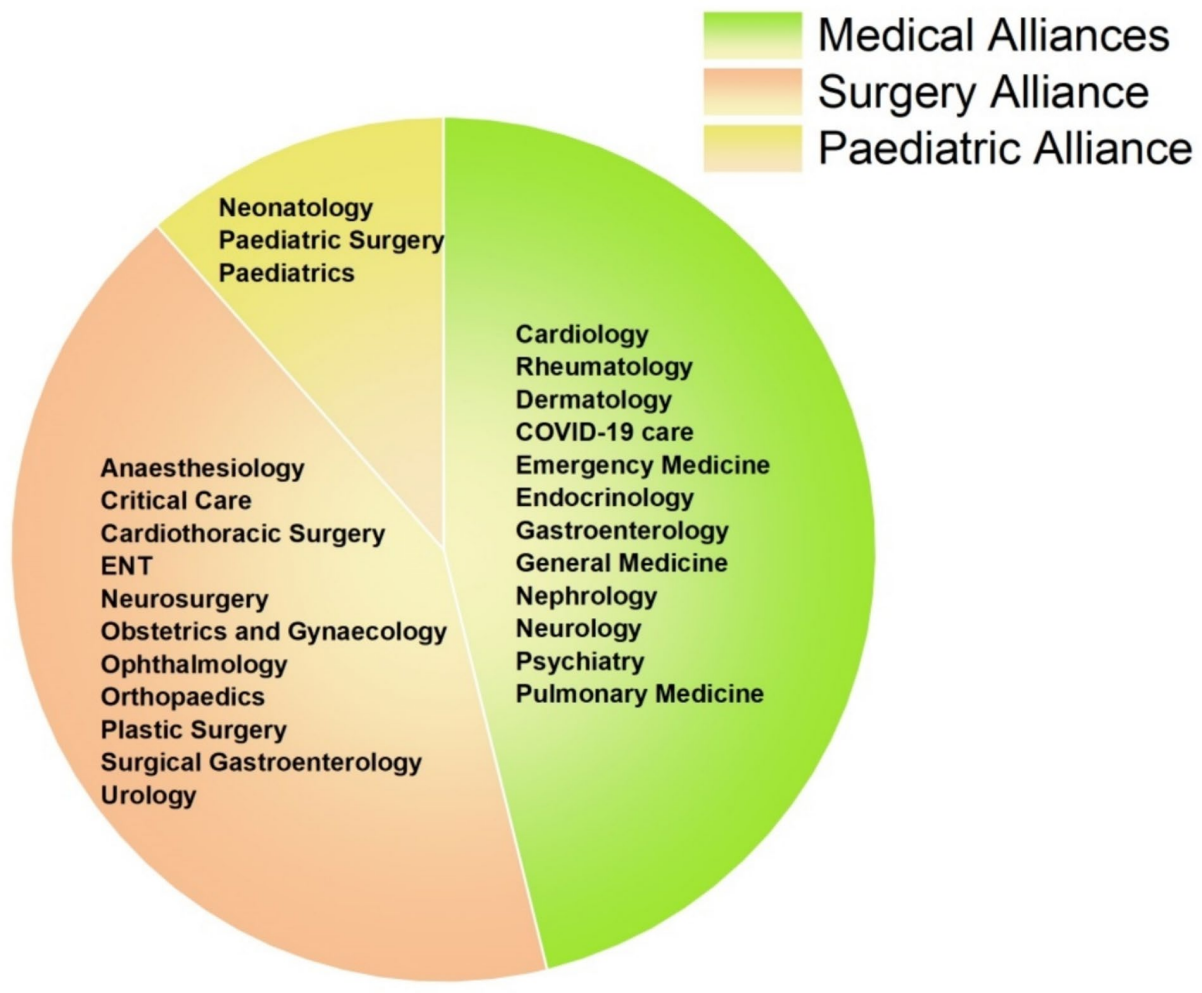
Distribution of hospital departments across clinical alliances. The pie chart categorizes hospital departments into three clinical alliances: Medical (green), Surgical (orange), and Paediatric (yellow). Each segment represents the departments contributing to their respective alliance for the analysis of antimicrobial resistance patterns.

Across hospital alliances (Figure 3), the Surgery Alliance consistently showed the highest resistance rates for all six pathogens. For instance, carbapenem resistance in *P. aeruginosa, A. baumannii*, and *Proteus* spp. exceeded 60% in this alliance. In contrast, the Paediatric Alliance demonstrated minimal resistance across all organisms (Table 2). These trends emphasize the importance of implementing targeted antimicrobial stewardship, particularly within surgical departments where resistance is most pronounced.

**Table 2:** Carbapenem resistance rates for various GNB across medical, surgery, and paediatric alliances, with the surgery alliance showing higher resistance.

Organisms (n)	MA (*n*)	MA (R %)	SA (*n*)	SA (R %)	PA (*n*)	PA (R %)
*E. coli* (433)	206	47.5	223	**51.5**	4	0.92
*K. pneumoniae* (733)	358	48.8	371	**50.6**	4	0.54
*P. aeruginosa* (290)	112	38.6	178	**61.3**	0	0
*A.baumannii* (388)	137	35.3	249	**64.1**	2	0.5
*Proteus* Spp. (126)	34	26.9	92	**73.1**	0	0
*Citrobacter* Spp. (37)	17	45.9	20	**54.1**	0	0

MA, Medical Alliance; SA, Surgery Alliance; PA, Paediatric Alliance; R, Resistance. Bold values indicate the highest percentage of resistance (R %) observed for each organism across the three hospital alliances. Bold values indicate the highest percentage of resistance (R %) observed for each organism across the three hospital alliances.

Interestingly, when comparing resistance patterns between IPM and MEM, specific differences were observed. In *Proteus* spp., 33.4% of respiratory isolates were resistant to IPM but remained sensitive to MEM. A similar pattern was noted in *Citrobacter* spp., where 16.6% of respiratory isolates showed resistance exclusively to IPM. In contrast, no substantial differences between the two drugs were observed in *E. coli, K. pneumoniae*, or *A. baumannii*.

### 3.3 Gender-wise distribution of carbapenem-resistant organisms

The gender distribution of CROs was analysed among the isolates (Table 3). The data showed that out of 2,007 total isolates, 716 (35.7%) were from females and 1,291 (64.3%) were from males. The highest number of carbapenem-resistant isolates were found in *K. pneumoniae* with 733 total isolates, comprising 238 females and 495 males, showing a significant gender difference [*χ*^2^(df) = 32.74, *p* < 0.001**]. *A. baumannii* also showed a high resistance rate with 388 total isolates, where 137 were from females and 251 from males. Other organisms such as *P. aeruginosa* had 290 total isolates (81 females, 209 males), *E. coli* had 433 total isolates (201 females, 232 males), *Proteus* spp. had 126 total isolates (45 females, 81 males), and *Citrobacter* spp. had 37 total isolates (14 females, 23 males). This data highlights the higher prevalence of carbapenem resistance in male patients compared to female patients.

**Table 3:** Gender-wise distribution of CROs among isolates, showing a higher prevalence in male patients compared to female patients.

Organism	Female	Male	Total	*χ*^2^ (df)	*p-*value
*A. baumannii*	137	251	388	32.74	<0.001**
*Citrobacter* Spp.	14	23	37		
*E. coli*	201	232	433		
*K. pneumoniae*	238	495	733		
*Proteus* Spp.	45	81	126		
*P. aeruginosa*	81	209	290		
Total	716	1,291	2007		

A total of 2007 valid cases were included in the analysis. The chi-square test results indicate a statistically significant association between gender and bacterial species distribution (*p* < 0.001).

### 3.4 Age-wise distribution of carbapenem-resistant organisms

**Figure 4:**
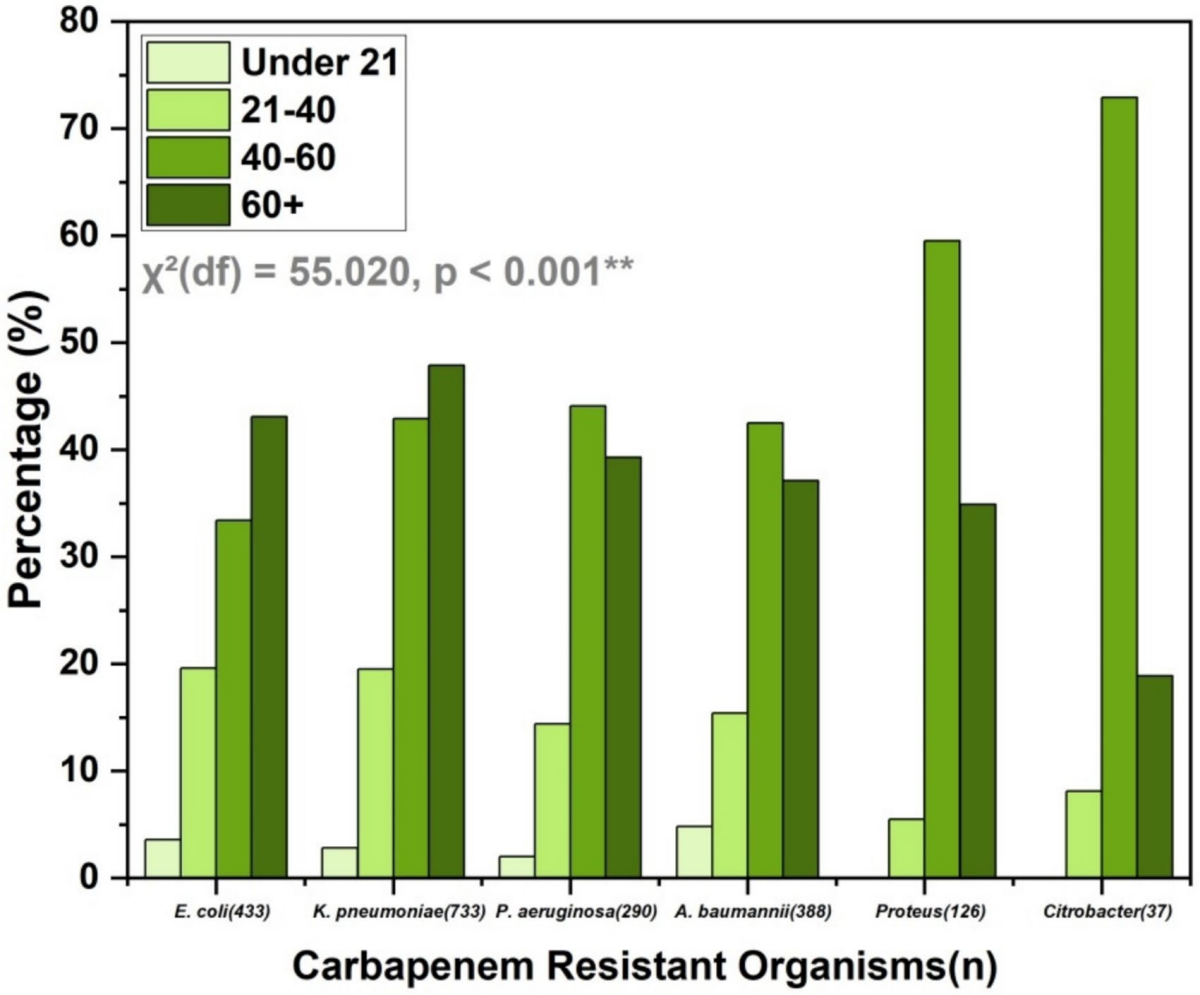
Age distribution of CROs, highlighting higher prevalence in individuals over 60 years, with significant age-related differences in prevalence. The chi-square test results indicate a statistically significant association between age groups and bacterial species distribution (*p* < 0.001).

The age distribution of CROs shows a significant age-related difference in prevalence [*χ*^2^(df) = 55.020, *p* < 0.001**]. The highest prevalence of carbapenem-resistant *E. coli* and *K. pneumoniae* is in individuals over 60 years, accounting for 43.1 and 47.9%, respectively, (Figure 4). Similarly, *P. aeruginosa* and *A. baumannii* are most common in the 40–60 and over 60 age groups. Proteus shows a markedly high prevalence in the 40–60 age group (59.5%), while Citrobacter is predominantly found in the 40–60 age group (72.9%). This data underscores the higher prevalence of CROs in older age groups, particularly those over 60 years old.

### 3.5 Quarterly trends in resistance patterns

The quarterly analysis of carbapenem resistance from July 2022 to June 2024 reveals distinct temporal and seasonal variations among key Gram-negative organisms (Table 4). The periods were categorized based on regional seasonal patterns in Tamil Nadu: *Southwest Monsoon (July–September), Northeast Monsoon (October–December), Dry Season (January–March), and summer (April–June)*. In the initial quarter (*Southwest Monsoon, July–September 2022*), *A. baumannii* exhibited the highest resistance rate at 46%, while *Citrobacter* spp. had the lowest at 1.5%, with a significant chi-square value [*χ*^2^(df) = 55.95, *p* < 0.001] (Supplementary Table 1).

**Table 4:** Quarterly prevalence of carbapenem resistance in Gram-negative organisms from July 2022 to June 2024.

Time period	*E. coli*	*K. pneumoniae*	*P. aeruginosa*	*A. baumannii*	*Proteus*	*Citrobacter*	*χ^2^* (df)	*p-*value
July–September 2022	11.9	21.3	11.8	46	14.2	1.5	**55.95**	<0.001**
October–December 2022	12.6	33.6	25.4	40.7	31.4	38.8		
January–March 2023	**14.4**	**51.9**	21.9	**76.3**	16.6	12.1		
April–June 2023	12.8	42.5	23.5	44	21.9	17.9		
July–September 2023	12.1	40.7	17.9	38.8	21.3	16.6		
October–December 2023	12.2	34.4	24	50.3	28.7	5.4		
January–March 2024	10.8	**43.11**	**38.7**	**57.2**	19.7	5		
April–June 2024	11.3	43.9	26.9	43.6	36.6	9.3		
Mean-R	12.26	38.90	23.76	49.61	23.5	13.32		

Resistances from a total of 2007 (total carbapenem resistant isolates) valid cases were included in the analysis. The chi-square test results indicate a statistically significant association between months and bacterial species distribution (*p* < 0.001). Bold values indicate the highest percentages of *E. coli*, *K. pneumoniae*, *A. baumannii*, and *P. aeruginosa* isolated during the study period.

Throughout the study, *A. baumannii* consistently displayed high resistance, peaking at 76.3% during the Dry Season (January–March 2023) and averaging 49.61% across the 2 years. *K. pneumoniae* also showed notable resistance trends, with a mean resistance of 38.92%, peaking at 51.9% in January–March 2023. In contrast, *E. coli* maintained lower resistance rates, generally around 12%, with a mild increase to 14.4% in the same Winter/Dry period (January–March 2023). *P. aeruginosa* demonstrated considerable variability, ranging from 11.8% (July–September 2022) to a peak of 38.7% (January–March 2024), averaging 23.76% overall.

*Proteus* spp. showed fluctuating resistance, with an overall average of 23.8%, reaching a high of 36.6% during summer (April–June 2024). *Citrobacter* spp., despite exhibiting the lowest overall resistance, had sporadic surges, notably 38.8% during the Northeast Monsoon (October–December 2022), and an average of 13.32% across the study period. These findings highlight the dynamic and seasonal nature of carbapenem resistance, with notable peaks during the Dry Season, particularly in *A. baumannii, E. coli, P. aeruginosa* and *K. pneumoniae*. The highly significant chi-square result supports substantial inter-organism and temporal variability, underscoring the critical need for continuous antimicrobial surveillance and seasonally tailored infection control strategies in healthcare settings.

### 3.6 Resistance patterns to other antibacterial agents

**Figure 5:**
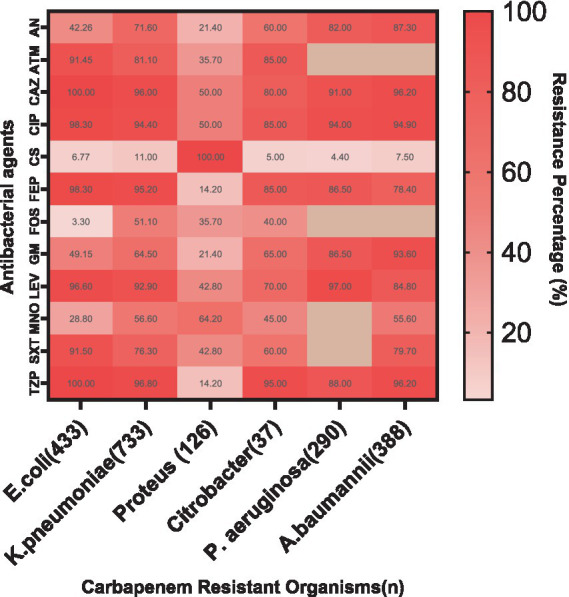
Resistance patterns of CROs to various antibacterial agents. The heat map illustrates high resistance levels in *E. coli*, *K. pneumoniae*, *P. aeruginosa*, and *A. baumannii* to multiple antibiotics, with lower resistance observed for Colistin, Minocycline and Fosfomycin. AN, Amikacin; ATM, Aztreonam; CAZ, Ceftazidime; CIP, Ciprofloxacin; CS, Colistin; FEP, Cefepime; FOS, Fosfomycin; GM, Gentamicin; LEV, Levofloxacin; MNO, Minocycline; SXT, Trimethoprim/Sulfamethoxazole; TZP, Piperacillin/Tazobactam.

The resistance patterns of CROs to various antibacterial agents reveal crucial insights (Figure 5). Notably, high resistance was observed in *E. coli* to Ceftazidime and Piperacillin/Tazobactam (both 100%), Ciprofloxacin (98.3%), and Cefepime (98.3%). *K. pneumoniae* also exhibited significant resistance to Ceftazidime (96%), Ciprofloxacin (94.4%), and Cefepime (95.2%). *P. aeruginosa* showed high resistance to Ciprofloxacin (94%) and Piperacillin/Tazobactam (88%), while *A. baumannii* exhibited considerable resistance to Ceftazidime (96.2%), Piperacillin/Tazobactam (96.2%), and Ciprofloxacin (94.9%).

In contrast, *Citrobacter* showed variable resistance patterns, with moderate resistance levels to Ceftazidime and Ciprofloxacin. *Proteus* also exhibited notable resistance, particularly to Ciprofloxacin and Piperacillin/Tazobactam (Supplementary Table 2).

Colistin remains effective against most strains, with notably low resistance in *E. coli* (6.77%), although *A. baumannii* showed some resistance (7.5%). Gentamicin and Levofloxacin showed varying levels of resistance across different organisms, with *E. coli* showing moderate resistance to Gentamicin (49.15%) and *K. pneumoniae* showing substantial resistance to Levofloxacin (92.9%). This data underscores the critical need for ongoing monitoring and judicious use of these antibiotics to manage and treat infections caused by CROs effectively.

### 3.7 Genotypic distribution

**Figure 6:**
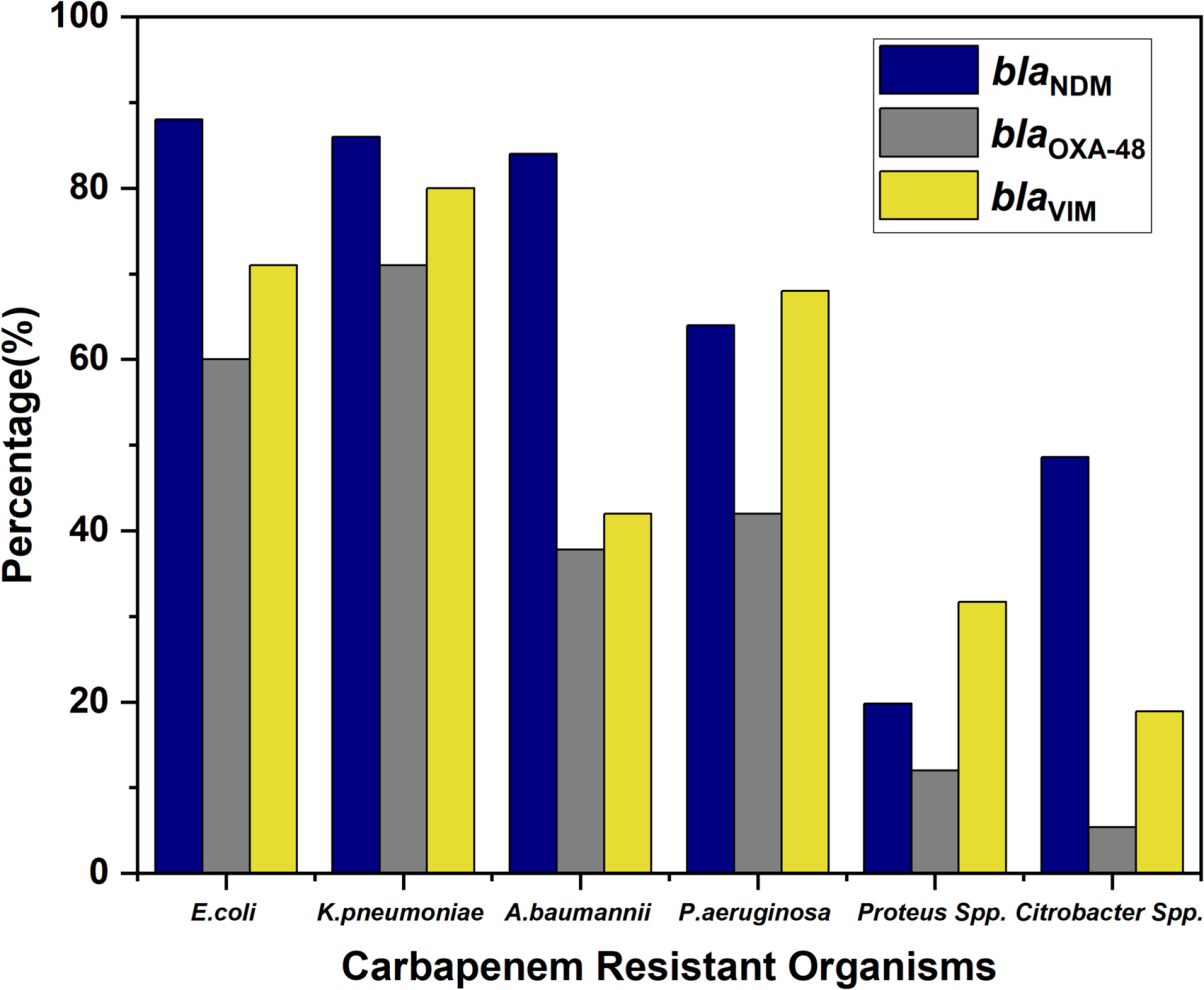
Distribution of carbapenemase genes (*bla*_NDM_*, bla*_OXA-48_*, and bla*_VIM_) across different bacterial isolates.

*Bla*_NDM_, *bla*_OXA-48_, and *bla*_VIM_ were highly prevalent among CR-GNB, especially in *E. coli*, *K. pneumoniae*, and *A. baumannii*, with lower rates in *Proteus* and *Citrobacter* spp. (Figure 6).

Importantly, co-carriage of multiple resistance genes was observed among several isolates. A total of 46% of isolates harbored all three genes (*bla*_NDM_ *+ bla*_OXA-48_ *+ bla*_VIM_), indicating a high level of genetic resistance complexity. Additionally, 9% of the isolates co-harbored *bla*_NDM_ *+ bla*_VIM_, while 12% carried *bla*_NDM_ *+ bla*_OXA-48_. The presence of these combinations underscores the evolving nature of carbapenem resistance and the potential for horizontal gene transfer among MDR organisms.

Organisms isolated from respiratory samples (medical alliance) showed the highest prevalence of all three resistance genes across all six bacterial species, highlighting the respiratory tract as a key reservoir for MDR strains. In *K. pneumoniae* and *A. baumannii*, blood from surgery alliance was the second most common source, while *Citrobacter* spp. showed higher gene prevalence in exudates. Notably, *P. aeruginosa* exhibited significant resistance gene carriage in urine samples, whereas for other organisms, urine was the least common source of resistance genes. No statistically significant association was observed with gender, age, and MIC values, highlighting that the distribution of these resistance genes is independent of patient demographics.

## 4 Discussion

Our investigation into the 2-year prevalence of carbapenem resistance among *E. coli*, *K. pneumoniae*, *Citrobacter*, *A. baumannii*, *Proteus*, and *P. aeruginosa* isolates in a tertiary care hospital in South India reveals critical insights into the ongoing challenge of antibiotic resistance in healthcare settings. Consistent with findings from other regions in India and globally, our study underscores the high prevalence of carbapenem resistance, particularly among *Klebsiella* and *Acinetobacter* species, which are notorious for their ability to acquire and disseminate resistance mechanisms (11). These resistance patterns can be compared with similar studies conducted globally, including the recent study by Abu Hammour et al. (12) in Jordan. A study conducted by Nieto-Saucedo also indicates a similar trend of resistance in *A. baumannii* and *K. pneumoniae* (13).

The resistance patterns observed in our study mirror those reported in other Indian regions, including North India, where similar high resistance rates have been documented (14). This nationwide issue highlights the urgent need for a coordinated and comprehensive approach to antimicrobial stewardship across the country. On a global scale, our data align with reports from regions with intense antibiotic pressure, such as Southeast Asia (15) and parts of Europe, further emphasizing the widespread nature of this public health threat (16).

Our study identified that carbapenem-resistant *E. coli*, *K. pneumoniae*, *A. baumannii*, *P. aeruginosa*, *Citrobacter*, and *Proteus* were isolated from various clinical specimens, including exudates, blood, respiratory specimens, and urine. Notably, *E. coli* and *K. pneumoniae* were predominantly found in urine samples, while *A. baumannii* and *P. aeruginosa* were more frequently isolated from respiratory specimens. *Citrobacter* and *Proteus* were also prevalent in urine samples. Changes in sample-wise distribution were observed when compared to findings from other studies (17, 18). These variations may be attributed to differences in patient demographics, hospital settings, sample collection practices, and regional prevalence of specific pathogens. Additionally, the selection pressure exerted by antibiotic usage patterns in different regions could also contribute to these differences in sample-wise distribution.

One of the most compelling aspects of this study is the observation of quarterly fluctuations in carbapenem resistance rates. These temporal variations offer a novel perspective on resistance epidemiology, suggesting that resistance trends are dynamic rather than static. Such patterns indicate that multiple external and internal factors may influence the rise and fall of resistance over time. Seasonal variations in antibiotic prescribing practices—particularly during periods such as the monsoon season when infectious diseases tend to spike—could contribute to increased antibiotic usage, thereby exerting selective pressure that promotes resistance. Additionally, modifications in hospital admission rates, changes in infection control protocols, and shifts in patient demographics may all play a role in shaping these fluctuations. For instance, an influx of critically ill patients requiring broad-spectrum antibiotics or the implementation of new antibiotic stewardship measures during certain quarters could significantly alter resistance patterns. Similar temporal dynamics have been reported in regional studies, such as that by Modi C. in Gujarat (19), underscoring the need for continuous, time-sensitive surveillance to better understand and respond to emerging resistance trends (19, 20).

In our study, we observed a significant difference in resistance patterns between IPM and MEM among the CROs. These findings suggest varying efficacy of these two carbapenems against the resistant strains, which may be attributed to differences in their molecular structure, permeability, or affinity for penicillin-binding proteins (PBPs). This disparity highlights the need for tailored antibiotic stewardship strategies when selecting carbapenem agents for empirical therapy, considering the specific resistance profiles observed. Results of a recent study conducted by Ikenoue et al. (21) positively correlate with this difference.

Our analysis revealed that the male-to-female ratio in the resistant isolates was skewed toward males, which aligns with other studies suggesting gender-based differences in susceptibility to infections or healthcare-seeking behavior. Similar results have been observed in studies conducted by Wang et al. (22). Furthermore, the age-wise distribution of resistance in our study showed a higher prevalence in older adults, particularly those over 60. This age group is often more vulnerable to infections due to comorbidities and frequent hospitalizations, making them more likely to be exposed to resistant pathogens. This observation is consistent with Zhang et al. (23) (China), who found the highest rates among individuals aged 65–79 years, and is supported by the CRACKLE study, which reported a median age of 70 years for carbapenem-resistant *K. pneumoniae* infections. However, a different study identified a significant proportion (48.30%) of carbapenem-resistant infections in the 36–65 age group, with lower prevalence in those aged 66–95 years and 0–33 years (24). This discrepancy highlights regional or methodological variations in age-related resistance patterns and underscores the importance of context-specific analyses in understanding resistance dynamics.

In our study, the ward-wise distribution of CROs showed a higher prevalence in ICUs and surgical wards, reflecting the increased use of broad-spectrum antibiotics and the higher risk of nosocomial infections in these settings. This finding aligns with (25), where the majority of carbapenem-resistant isolates were obtained from general wards (41.5%) and ICUs (33.2%). It is also consistent with a study conducted by Kumari N, which found a significant proportion of carbapenemase-producing isolates in the medicine ICU (47.0%) and surgery ward (35.3%) (18). However, Nair and Vaz (26) reported that the majority of CRE isolates were found in hospitalized patients (42%), followed by OPD (32%) and ICU (26%). While our study and others consistently highlight ICUs as significant hotspots for resistance, the variation in distribution across different healthcare settings may be attributed to differences in antimicrobial stewardship practices, infection control protocols, patient case-mix, and institutional diagnostic approaches.

In addition to carbapenem resistance, our study also documented the resistance profiles of these isolates to other commonly used antibiotics. The data revealed a high degree of MDR, with many CROs also showing resistance to cephalosporins, aminoglycosides, and fluoroquinolones. Our findings align with previous studies (27, 28), which reported high levels of MDR among carbapenem-resistant isolates. Similar to these studies, we observed resistance to cephalosporins, aminoglycosides, and fluoroquinolones. While specific resistance rates varied, the consistent trend across studies highlights the urgent need for alternative therapeutic strategies to combat these extensively drug-resistant pathogens.

In our study, the distribution of carbapenemase genes among GNB revealed a concerning trend, with a notably high prevalence of *bla*_NDM_*, bla*_OXA-48_*, and bla*_VIM_ genes across several species. *E. coli* and *K. pneumoniae* exhibited particularly high rates of these genes, with *E. coli* showing 88% *bla*_NDM_, 60% *bla*_OXA-48_, and 71% *bla*_VIM_, while *K. pneumoniae* displayed 86, 71, and 80%, respectively. This highlights the growing threat posed by MDR Enterobacteriaceae, which are known to be efficient in acquiring and disseminating resistance determinants (5). The co-existence of these genes significantly compromises therapeutic efficacy, often leaving limited treatment options such as polymyxins or tigecycline, which themselves are associated with toxicity and emerging resistance.

Among non-fermenters, *A. baumannii* showed an 84% prevalence of *bla*_NDM_ and moderate levels of *bla*_OXA-48_ (37.8%) and *bla*_VIM_ (42%), suggesting its expanding role in healthcare-associated infections with a multidrug-resistant phenotype. *P. aeruginosa* also carried all three genes at appreciable levels, further compounding its known intrinsic resistance mechanisms. Though *Proteus* spp. and *Citrobacter* spp. had comparatively lower frequencies, the presence of *bla*_NDM_ in 48.6% of *Citrobacter* spp. and the detection of all three genes even in *Proteus* spp. point toward the silent spread of these genes among lesser-monitored species (29, 30).

Of particular concern is the co-occurrence of multiple resistance genes within the same isolate, as seen in 46% of cases (NDM + VIM + OXA-48), 9% (NDM + VIM), and 12% (NDM + OXA-48). These combinations enhance the spectrum and level of resistance, potentially leading to complete therapeutic failure. Such MDR not only limits the choice of antimicrobials but also increases morbidity, mortality, and healthcare costs. The presence of multiple carbapenemase genes in a single isolate also raises the possibility of horizontal gene transfer through plasmids, accelerating the spread of resistance within hospital environments (5). This underlines the urgent need for robust molecular surveillance, strict antimicrobial stewardship, and effective infection control measures to mitigate the clinical and epidemiological impact of these formidable pathogens.

## 5 Strengthening hospital infection control in response to CRO trends

As an impact of this study, several targeted interventions were implemented within the hospital to strengthen infection prevention and control (IPC) practices and mitigate the spread of CROs. Active surveillance cultures were initiated in high-risk units such as ICUs and surgical wards to facilitate early detection of CRO colonization. Isolation precautions were reinforced for affected patients, with dedicated staff and cohorting strategies to minimize cross-transmission. Periodic audits and feedback sessions were introduced to monitor antibiotic prescribing patterns, ensuring stricter adherence to antimicrobial stewardship protocols. Hand hygiene practices were re-emphasized through staff training, compliance monitoring, and real-time feedback systems. Environmental disinfection procedures were also upgraded, incorporating enhanced terminal cleaning with sporicidal agents and UV-based disinfection in critical care areas. Additionally, laboratory reports for isolates carrying multiple carbapenemase genes were flagged with clinical alerts, enabling timely and appropriate infection management. Collectively, these measures contributed to a more vigilant and proactive IPC environment within the healthcare facility.

## 6 Conclusion

This 2-year surveillance study highlights the alarming burden of carbapenem resistance among GNB, particularly in *K. pneumoniae*, *A. baumannii*, and *E. coli*, in a tertiary care hospital setting in South India. The high prevalence of resistance to multiple antibiotic classes, along with the co-existence of major carbapenemase genes (*bla*_NDM_, *bla*_OXA-48_, and *bla*_VIM_), reflects the growing challenge posed by MDR organisms in clinical practice. The study also revealed significant variations in resistance trends based on sample type, patient age, gender, and hospital wards. Notably, the quarterly fluctuations in resistance patterns suggest a dynamic and evolving resistance profile, underscoring the importance of continuous, time-based monitoring. The widespread distribution of carbapenemase genes across various species, including lesser-monitored organisms like *Citrobacter* and *Proteus*, calls for enhanced molecular diagnostics and routine surveillance.

In conclusion, the findings reaffirm the critical need for ongoing surveillance, rational antibiotic use, and targeted research to address the threat of antimicrobial resistance. These insights contribute meaningfully to the broader understanding of carbapenem resistance patterns in healthcare settings and provide a foundation for future investigations aimed at developing effective containment strategies.

## 7 Limitations and future directions

This single-center study limits generalizability due to localized antimicrobial practices. Molecular analysis was restricted to gene detection without assessing expression levels. Environmental or healthcare worker surveillance was not performed. Additionally, data on prior antibiotic exposure and patient comorbidities were incomplete, limiting correlation analyses. Lack of data on regional antibiotic policies has also been acknowledged as a limitation in this study, as it restricts broader interpretation of resistance trends. Future studies should include multi-center surveillance, whole-genome sequencing, longitudinal monitoring, environmental sampling, and evaluation of targeted antimicrobial stewardship interventions.

## Data Availability

The original contributions presented in the study are included in the article/Supplementary material, further inquiries can be directed to the corresponding authors.
