# Supplementary material for: Prevalence and molecular insights into carbapenem resistance: a 2-year retrospective analysis of superbugs in South India
Source: Front Med (Lausanne). 2025 May 30;12:1571231. doi: 10.3389/fmed.2025.1571231 (PMC12162471; doi:10.3389/fmed.2025.1571231)
Supplement: Supplementary file 1 [file Data_Sheet_1.pdf]

Supplementary Table.1 Sample-wise, organism-wise and season wise quarterly resistance to Imipenem and Meropenem from july 2022 to july 2024 in detail.

Organism	Sample	July-Sep 2022 (Southwest Monsoon)		Oct-Dec 2022 (Northeast Monsoon)		Jan-Mar 2023 (Dry Season)		Apr-Jun 2023 (Summer)		Jul-Sep 2023 (Southwest Monsoon)		Oct-Dec 2023 (Northeast Monsoon)		Jan-Mar 2024 (Dry Season)		Apr-Jun 2024 (Dry Season)	
		IMP-R(%)	MER-R(%)	IMP-R(%)	MER-R(%)	IMP-R(%)	MER-R(%)	IMP-R(%)	MER-R(%)	IMP-R(%)	MER-R(%)	IMP-R(%)	MER-R(%)	IMP-R(%)	MER-R(%)	IMP-R(%)	MER-R(%)
	Blood	14.2	14.2	7.1	7.1	7.1	7.1	13.8	16.6	23.5	20.5	0	2.1	2.8	5.7	15.1	15.1
	Urine	10.2	10.2	11.8	12.2	13.6	13.6	9.8	10.5	9	9.3	10.3	9.9	9.2	9.2	7.9	8.4
	Respiratory	33.3	33.3	14.2	14.2	0	0	18.1	18.1	12.5	12.5	11.7	17.6	25	25	0	0
	Exudates	17.2	16	15.4	14.4	18.8	18.8	7.6	15.3	18.2	18.2	23.1	23.9	16.9	18.5	20.5	19.6
**E.coli**	**Total**	**11.7**	**11.9**	**12.6**	**12.6**	**14.4**	**14.4**	**11.4**	**12.8**	**12.1**	**12.1**	**12.1**	**12.2**	**10.8**	**10.8**	**11.1**	**11.3**
	Blood	19.9	25	33.3	41.6	35.7	50	30	45	52	56	39.2	42.8	55.5	55.5	73.6	73.6
	Urine	37.5	19.8	28.1	29.5	39.5	54.1	33.7	40.4	23.6	26.8	25.7	28.1	23.7	27.7	33.3	34.3
	Respiratory	17.1	20	30.5	30.5	31.8	59	41.1	47	50	54	36.8	38.5	51.6	48.3	51.3	48.6
	Exudates	20.4	24.4	37.5	37.5	36.3	47.7	41	42.4	43.2	43.2	37.2	37.2	50.8	51.6	44.3	46.3
**K.pneumoniae**	**Total**	**19.9**	**21.3**	**32.6**	**33.6**	**36.4**	**51.9**	**37**	**42.5**	**38.4**	**40.7**	**32.9**	**34.4**	**42.1**	**43.11**	**43.1**	**43.9**
	Blood	22.2	22.2	43	43	80	60	0	0	25	25	37.5	37.5	33.3	33.3	37.5	37.5
	Urine	11.1	11.1	21.7	21.7	57.1	28.5	45	35	17.3	17.3	22.5	17.5	34.4	31	10.5	5.2
	Respiratory	81.82	75.76	66.6	66.6	87	87	68	68	61.5	61.5	85.29	85.2	72.2	72.2	86.9	86.9
	Exudates	54.3	54.29	43.3	43.3	77.7	72.2	32.3	32.3	39.2	39.2	50	50	62.5	62.5	36.5	40.3
**A.baumannii**	**Total**	**46**	**43.36**	**40.7**	**40.7**	**76.3**	**65.7**	**44**	**42.1**	**38.8**	**38.8**	**50.3**	**48.9**	**57.2**	**56.5**	**42.7**	**43.6**
	Blood	0	0	40	20	0	0	0	0	12.5	0	0	0	33.3	33.3	6.6	6.6
	Urine	26	23.9	31.4	25.7	44	48	31.9	34	28.8	28.8	26.9	23	41	38.4	43.8	38.5
	Respiratory	16.6	16.6	16.6	8.3	0	0	20	20	13	13	21.6	21.6	47	50	38.4	38.4
	Exudates	12.3	12.3	22.7	10.6	10.5	10.5	19.17	17.8	14.4	14.4	24.7	23.8	35	29.8	17.4	15.11
**P.aeroginosa**	**Total**	**11.8**	**10.4**	**25.4**	**14.4**	**20.5**	**21.9**	**23.5**	**23.5**	**17.9**	**17.3**	**24**	**22.5**	**38.7**	**35.8**	**26.9**	**23.9**
	Blood	0	0	0	0	100	0	0	0	50	100	0	0	0	0	0	0
	Urine	16.6	16.6	20	13.3	12.5	0	20	20	20	13.3	23.5	0	14.2	0	37.5	0
	Respiratory	0	0	0	0	0	0	100	100	50	0	66.6	16.6	0	0	100	0
	Exudates	13.3	6.6	36.8	15.7	15	5	18.6	5	19	4.7	27.1	2.8	22.4	6.1	36.5	4.8
**Proteus**	**Total**	**14.2**	**8.5**	**31.4**	**14.8**	**16.6**	**3.3**	**21.9**	**12.19**	**21.3**	**9.8**	**28.7**	**3.1**	**19.7**	**4.2**	**36.6**	**3.3**
	Blood	0	0	0	0	0	0	0	0	0	0	0	0	0	0	0	0
	Urine	0	0	4.3	4.3	0	0	0	0	3.2	3.2	0	0	0	0	12.5	12.5
	Respiratory	0	0	0	0	100	100	0	0	0	0	0	0	0	0	0	0
	Exudates	4	4	23	19.2	25	16.6	31.5	36.8	40	35	14.2	10.7	14.2	14.2	0	0
**Citrobacter**	**Total**	**1.5**	**1.5**	**38.8**	**33.3**	**12.1**	**9**	**15.3**	**17.9**	**16.6**	**14.8**	**5.4**	**4**	**5**	**5**	**9.3**	**9.3**

Supplementary Table.2: Antimicrobial susceptibility (%) of gram-negative bacterial isolates to various antibiotics.

This table summarizes the percentage of susceptibility of six major Gram-negative organisms—*E. coli*, *K. pneumoniae*, *Proteus* spp., *Citrobacter* spp., *P. aeruginosa*, and *A. baumannii*—to commonly used antibiotics, including aminoglycosides, β-lactams, carbapenems, fluoroquinolones, and others. The data reflect resistance trends observed in the study population.

Organisms	AN	ATM	CAZ	CIP	CS	FEP	FOS	GM	LEV	MNO	SXT	TZP
E.coli(433)	42.26	91.45	100	98.3	6.8	98.3	3.3	49.2	97	28.8	91.5	100
K.pneumoniae (733)	71.6	81.1	96	94.4	11	95.2	51.1	64.5	93	56.6	76.3	96.8
Proteus (126)	21.4	35.7	50	50	100	14.2	35.7	21.4	43	64.2	42.8	14.2
Citrobacter(37)	60	85	80	85	5	85	40	65	70	45	60	95
P. aeruginosa(290)	82		91	94	4.4	86.5		86.5	97			88
A.baumannii(388)	87.3		96.2	94.9	7.5	78.4		93.6	85	55.6	79.7	96.2
